# Effect of Fixation Methods on Biochemical Characteristics of Green Teas and Their Lipid-Lowering Effects in a Zebrafish Larvae Model

**DOI:** 10.3390/foods11111582

**Published:** 2022-05-28

**Authors:** Maoyun Li, Lulu Guo, Ruixue Zhu, Dongmei Yang, Yue Xiao, Yanping Wu, Kai Zhong, Yina Huang, Hong Gao

**Affiliations:** 1 College of Biomass Science and Engineering, Sichuan University, Chengdu 610065, China; myleecheer@163.com (M.L.); eric211@163.com (K.Z.); gao523@hotmail.com (H.G.); 2 West China School of Public Health, Sichuan University, Chengdu 610065, China; boiler_lu@163.com (L.G.); yangdm8365@163.com (D.Y.); scu_xyue@163.com (Y.X.); hyn427@scu.edu.cn (Y.H.); 3 Sichuan Calfstone Analysis Co., Ltd., Chengdu 610052, China; zhu_ruixue@163.com

**Keywords:** pan-fire, steam, tea quality, chemical compounds, hypolipidemic effect

## Abstract

Fixation is a key process contributing to different flavors of green tea and pan-fire and steam were the common fixation methods applied conventionally. In this study, pan-fired green tea (PGT) and steamed green tea (SGT) produced by different fixation methods were compared in characteristic biochemicals including volatile compounds, amino acids, catechins and alkaloids, together with evaluating their tastes and lipid-lowering effects. PGT and SGT could be distinguished clearly by orthogonal partial least squares discriminant analysis (OPLS-DA) and heatmap. SGT had higher contents of volatile alcohols (44.75%) with green and floral attributes, while PGT had higher contents of volatile esters (22.63%) with fruity and sweet attributes. Results of electronic tongue analysis showed that PGT and SGT had similar taste of strong umami and sweetness, but little astringency and bitterness. In addition, amino acids were more abundant in PGT (41.47 mg/g in PGT, 33.79 mg/g in SGT), and catechins were more abundant in SGT (111.36 mg/g in PGT, 139.68 mg/mg in SGT). Zebrafish larvae high-fat model was applied to study the lipid-lowering effects of PGT and SGT. Results showed that both SGT and PGT had lipid-lowering effects, and the lipid level was decreased to 61.11 and 54.47% at concentration of 300 mg/L compared to high-fat group, respectively. Generally, different fixation methods of pan-fire and steam showed significant effects on aroma and contents of characteristic chemical compounds (amino acids and catechins) of green tea, but no effects on the taste and lipid-lowering activity.

## 1. Introduction

Green tea has become one of the most popular beverages in the world, which has pleasant aroma and mellow taste, together with health benefits including antioxidant, anti-bacterial, anti-cancer, anti-obesity and so on [1,2,3]. Numerous chemical components in green tea, such as volatile compounds, catechins, alkaloids and amino acids, are contributing to the aroma and taste of green tea. The varieties and contents of these chemical compounds are mainly affected by tea cultivar, growth environment, harvesting season and manufacturing process [4].

**Figure 1:**
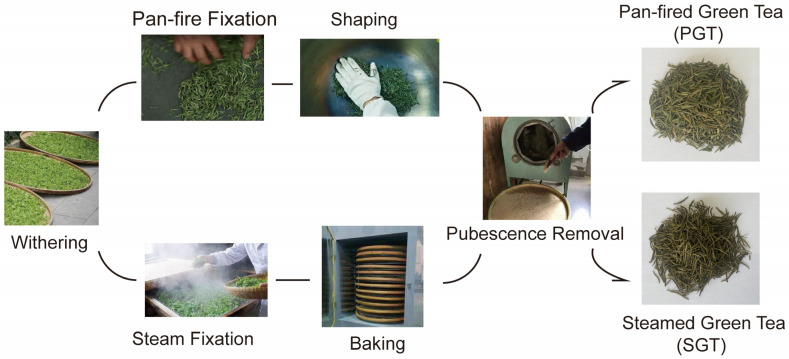
Manufacturing process of pan-fired green tea (PGT) and steamed green tea (SGT).

Fixation for deactivation of endogenous enzymes is regarded as the most important step of green tea manufacturing process [5]. Fixation methods include pan-fire, steam, microwave and hot air. Microwave fixation could retain chlorophyll and V_C_, improve the tea infusion color, but lead to the tea aroma and taste poorer [6]. Hot air fixation could keep the color and aroma of tea leaves but make the edge of the leaves dry due to the high temperature [7]. For de-enzyme, pan-fire and steam treatments are conventionally applied in Guizhou Province of China [8]. As shown in Figure 1, Guizhou pan-fired green tea (PGT) is produced through withering, pan-fire fixation, shaping and pubescence removal, and Guizhou steamed green tea (SGT) is produced through withering, steam fixation, baking and pubescence removal. These two green teas are the main tea products in Guizhou Province and widely favored by consumers. PGT with long fixation time was described as ‘roasted’ taste and ‘chestnut-like’ aroma attributes, while SGT with short fixation time was regarded as a ‘green’ note attribute [8,9]. To date, research on the differences of PGT and SGT mainly focused on the aroma analysis. Wang et al. [10] determined aroma composition according to GC/MS and reported that the aroma concentrations in SGT (4168–10706 μg/L) were significantly higher than those in PGT (959–2608 μg/L). Han et al. [8] found that pan-fired ‘Bai-Sang Cha’ had significantly higher levels of catechins, linalool and its derivatives, geraniol and β-ionone and lower level of indole. Few studies have been paying attention to analyze the overall differences in characteristic biochemical compounds and the lipid-lowering effect between PGT and SGT.

With the prevalence of overweight conditions and obesity which could be induced by a high-fat diet [11], lipid-lowering effect of green tea has been getting more and more attention. Green tea and its extract have been proved to prevent obesity in epidemiological studies, clinical trials and cellular and animal experiments [1,12,13], but the research about the effect of different fixation methods on lipid-lowering activity of green tea is scarce. Compared to other animal models, zebrafish (*Danio rerio*) is an excellent model organism to study lipid metabolism in a short time, which has the advantages of small size, high reproducibility, short life cycle and transparent body of larvae. Previous studies had verified the feasibility of zebrafish larvae as a high-fat model and the lipid-lowering effects of green tea and dark tea extracts [4,14].

This study aimed to investigate the effect of pan-fire and steam fixation on the aroma, taste and lipid-lowering activity of green tea. Chemical components including aroma volatile compounds, catechins, amino acids and alkaloids were determined, together with the representation of taste using an electronic tongue system and evaluation of lipid-lowering effect using a high-fat zebrafish larvae model. This study provides insights into how different fixation methods contribute to the quality characteristics of green tea and expects to provide a theoretical reference and objective basis for the directional processing of high-quality green tea.

## 2. Materials and Methods

### 2.1. Materials

PGT and SGT were obtained from Guizhou Gengtian Modern Agriculture Investment and Development Co. Ltd. (Guizhou, China), which were produced using a bud with a leaf (*Camellia sinensis* cv. *Qianmei* 601) harvested from March to April in 2020 as raw material. The production processes of PGT and SGT were conducted according to the Guizhou provincial standard of processing technical specification of Meitan Cuiya tea (DB 52/T 1002-2015) and Guizhou green tea—technical specifications for Guizhou Zhen tea (DB 52/T 637-2010), respectively. PGT was produced through the following processes: withering (thickness of leaves, <8 cm; 4–8 h), pan-fire fixation (180–200 °C; 8–10 min; water content, 60%), shaping (80–90 °C; 15–20 min; water content, 40%) and pubescence removal (90–120 °C; water content, 6–8%). SGT was produced through the following processes: withering (thickness of leaves, 2–4 cm; 4–6 h), steam fixation (0.5–0.8 MPa; 200–240 °C; 7–8 s; water content, 70%), baking (60–70 °C; 1.2–2.0 h; water content, 7.0–7.5%) and pubescence removal (40 °C; 60–90 min). The finished green teas of PGT and SGT were ground using a model BO-150P1 grinder (Boou Hardware Product Co. Ltd., Zhejiang, China) and sifted through the 80 meshed per inch to carry on the following analysis.

Caffeine, theobromine, theophylline, gallic acid (GA), epicatechin (EC), epigallocatechin gallate (EGCG), epicatechin gallate (ECG), epigallocatechin (EGC), catechin (C) and *n*-alkane mixture (C_6_–C_24_) were purchased from Sigma-Aldrich Co. (St. Louis, MO, USA). Standard solution of amino acids was purchased from MembraPure GmbH Co. (Berlin, Germany). Chicken egg yolk powder (S30910, BR grade) was purchased from Shanghai Yuanye Biotechnology Co. Ltd. (Shanghai, China). Oil red O solution (ORO), 4% paraformaldehyde fixation and PBS solution were purchased from Beijing Solarbio Science & Technology Co. LTD (Beijing, China). All other chemicals and reagents were purchased from Chengdu Kelong Chemical Reagent Factory (Chengdu, China). Wild-type (AB strain) zebrafish were obtained from China Zebrafish Resource Center (Wuhan, China).

### 2.2. Analysis of Volatile Compounds

Volatile compounds were determined using the headspace solid-phase microextraction (HS-SPME) combined a QP2010 SE GC-MS (Shimadzu Co., Ltd., Kyoto, Japan) according to a method described previously [14]. Volatile compounds were separated with the RTX-5Sil MS capillary column (0.25 μm × 0.25 mm × 30 m, Shimadzu). The temperature of ion source and interface was 230 and 280 °C, respectively. Identification of volatiles was carried out by matching mass to NIST 14s MS data library and comparing retention index (RI) to *n*-alkanes (C_6_–C_24_). The content of volatile compounds was shown by the peak area percentage (%).

### 2.3. Determination of Amino Acids

The extraction and determination of free amino acids of tea samples were conducted according to the method in our laboratory [15]. Quantification of amino acids was conducted by external standard method and the contents were shown as mg/g tea.

### 2.4. Quantification of Gallic Acid, Catechins and Alkaloids

Gallic acid, catechins and alkaloids contents were determined following the method in our laboratory using a HPLC system [16]. HPLC analysis was carried out using an Agilent 1260 LC system (Agilent Technologies Inc., Santa Clara, CA, USA) equipped with a diode array detector (detection wavelength, 280 nm) coupled to an Inertisil ODS-3 (5 μm, 4.6 mm × 250 mm; GL-science Inc., Tokyo, Japan). External standard method was used to conduct quantification and the contents were shown as mg/g tea.

### 2.5. Taste Evaluation by Electronic Tongue

The sample preparation and electronic tongue analysis were conducted as described previously [17].

### 2.6. The Lipid-Lowering Effect Based on a Zebrafish Larvae Model

Evaluation of lipid-lowering effects of PGT and STG based on a zebrafish larvae model was based on the method described previously [4]. The water extracts of tea samples were used to evaluate the lipid-lowering effect of PGT and SGT. Tea powder (5 g) was added to 500 mL of boiling water, and the mixture was stirred in a boiling water bath for 30 min. Then, the mixture was filtered, and the filtrate was concentrated and freeze-dried to obtain water extract of tea samples.

All wild-type zebrafish of AB strain were raised and maintained at 28 °C under a 14 h light/10 h dark cycle. After natural mating, embryos were collected and incubated in fish water at 28 °C. After 5 days post-fertilization, larvae were collected and treated with 0.1% (*m*/*v*) egg yolk for 48 h to exhibit a high-fat diet (HFD) model. Then, high-fat zebrafish larvae were treated with tea water extract which dissolved in fish water for 48 h at a final concentration of 50, 100, 200 and 300 μg/mL, respectively. In addition, high-fat zebrafish larvae treated with fish water, simvastatin (0.04 μg/mL), DMSO (0.1%, *v*/*v*) for 48 h were set as HFD, positive control and solvent control, respectively. Zebrafish larvae without HFD treatment were set as control. Zebrafish larvae were placed in 6-well cell culture plates at a density of 15 zebrafish larvae in 6 mL of solution per group for treatment. Finally, all zebrafish larvae were fixed with 4% paraformaldehyde at 4 °C overnight and washed three times using 10 mM PBS solution. After that, all zebrafish larvae were dipped in 60% (*v*/*v*) isopropanol for 30 min to dehydrate, then dehydrated zebrafish larvae were dyed using 0.3% (*v*/*v*) ORO solution for 3 h in dark. After washing with 60% (*v*/*v*) isopropanol three times and then 10 mM PBS solution three times, zebrafish larvae were ready for microscopic observation using a Leica M205 FA stereomicroscope (Leica Ltd., Wetzlar, Germany) and images at a certain magnification were captured. The lipid level of zebrafish larvae was normalized to the integral optical density (IOD) values through the Image-Pro Plus software (v6.0, Media Cybernetic Inc., Rockville, MD, USA), and the lipid-lowering effect of the tea samples was evaluated based on the HFD group. The animal experiment was performed according to the guidelines of the Animal Care and Use Committee of Sichuan University (Chengdu, China) and approved by the institutional review board of the Medical Faculty at the West China Hospital, Sichuan University.

### 2.7. Statistical Analysis

Significant differences were determined by SPSS software (v22.0, SPSS Inc., Chicago, IL, USA) using student’s *t*-test. Orthogonal partial least squares discriminant analysis (OPLS-DA) was performed using SIMCA-P (v14.1, Umetrics AB, Umea, Sweden) and R (v4.1.0) was used to generate heatmap with a pheatmap package.

## 3. Results and Discussion

### 3.1. Effect of Fixation Methods on Volatile Profiles

**Figure 2:**
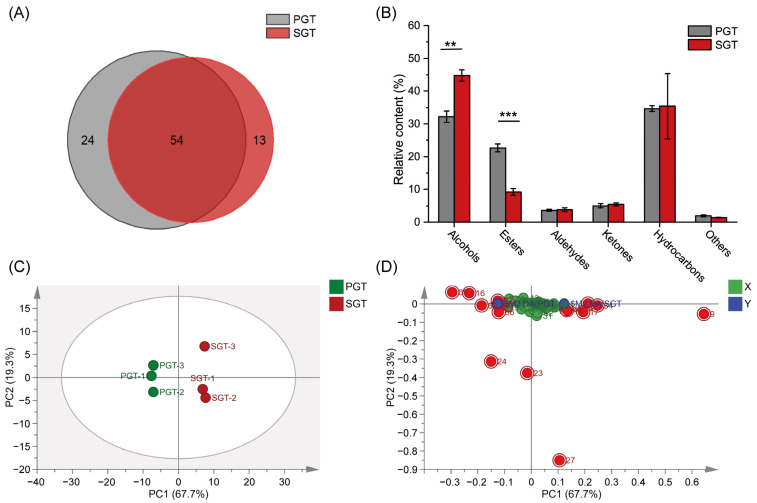
Effect of fixation methods on volatile compounds of green tea. (**A**) Venn diagram of volatile compounds; (**B**) Relative contents of volatile compounds in different classes; (**C**) Score scatter plot of OPLS-DA analysis; (**D**) Loading scatter plot of OPLS-DA analysis. PGT, pan-fired green tea; SGT, steamed green tea; OPLS-DA, orthogonal partial least squares discriminant analysis. ** *p* < 0.01; *** *p* < 0.001.

As shown in Table S1, 91 volatile compounds were detected in PGT and SGT, including 17 alcohols, 5 aldehydes, 26 hydrocarbons, 10 ketones, 28 esters and 5 others. As the Venn diagram (Figure 2A) shows, PGT and SGT shared 54 volatile compounds in common, which accounted for 79.69 and 82.78% in PGT and SGT, respectively. Moreover, PGT and SGT had 24 and 13 unique volatile compounds, respectively. The most abundant volatiles in PGT and SGT both were linalool (floral), _D_-limonene (fruity, lemon-like), geraniol (rose-like, sweet, honey-like) and 1,3,5,7-cyclooctatetraene [18], which accounted for 14.21, 9.33, 8.37 and 6.53% in PGT, respectively, and 23.7, 10.91, 4.93 and 6.24% in SGT, respectively.

As shown in Figure 2B, PGT had more volatile esters (*p* < 0.001), and SGT had more volatile alcohols (*p* < 0.01). In addition, volatile hydrocarbons were detected abundantly in both PGT and SGT. In SGT, high levels of alcohols such as linalool, geraniol, 1-hexanol, 5-methy-2-heptanol, 1-octen-3-ol, 1-octanol, *trans*-2-pinanol and cedrenol (*p* < 0.05), endowed SGT with characteristic green and floral odor notes [18]. The ‘green’ odor of tea is mainly attributed to C6 and C9 alcohols and aldehydes [19]. In PGT, high levels of esters such as ethyl hexanoate, *cis*-3-hexenyl acetate, (*E*)-3-hexen-1-yl butyrate, butyl hexanoate, ethyl caprylate and (*Z*)-3-hexen-1-yl caproate (*p* < 0.05), endowed PGT with characteristic fruity and sweet odor notes [20]. Most of volatile compounds were formed by the enzymatic, hydrolysis and thermal cracking reactions in the mechanical damage and high temperature of leaves during processing [9]. Many alcohol volatiles are reported to release from glycoside-bonded molecules by hydrolyzing during withering [21]. Relatively long time of pan-fire fixation may cause alcohols with low boiling point to volatilize and promote the formation of esters by esterification reaction of alcohols and acids. Cui et al. [20] have reported that the content of *cis*-3-hexenyl hexanoate increased after the fixation process of pan-fire, which may be formed from the esterification of *cis*-3-hexenol at high temperature.

Results of OPLS-DA analysis showed that PGT and SGT could be clearly distinguished from one another based on the relative contents of volatile compounds (Figure 2C), and the loading plot was shown in Figure 2D. The principal component 1 (PC1) and principal component 2 (PC2) of the OPLS-DA model could explain 67.7 and 19.3% of the total variance, respectively. Volatile compounds with VIP > 1 and *p* < 0.05 (Table S2) were considered as differential volatiles, and these compounds included linalool (floral), ethyl caprylate (fruity), azulene, geraniol (rose-like, sweet, honey-like), 1-hexanol (green, resin), cedrenol (woody), ethyl hexanoate (tinned apple), acenaphthylene, *trans*-2-pinanol, butyl hexanoate, acenaphthene, ethyl palmitate (fatty, rancid, fruity, waxy) and *cis*-3-hexenyl acetate (fruity, banana-like) [8,18,22,23,24]. Overall, green teas processed by different fixation methods of pan-fire and steam had different aroma attributes and volatile composition.

### 3.2. Effect of Fixation Methods on Taste Characteristics

**Figure 3:**
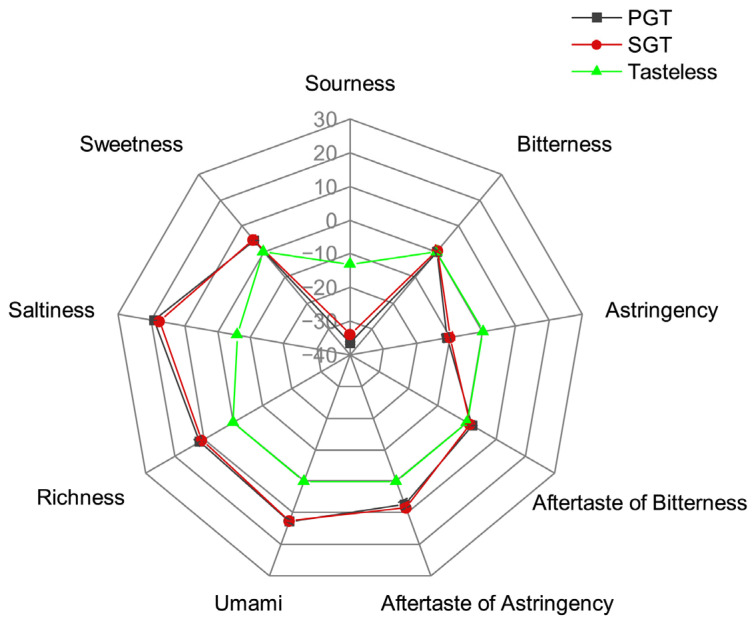
Effect of fixation methods on taste characteristics of green tea by electronic tongue analysis. PGT, pan-fired green tea; SGT, steamed green tea.

The electronic tongue system which is similar to human tongue was used to measure taste intensity of the tea infusion [17]. As shown in Figure 3, six of the taste attributes were measured in this study, including saltiness, sweetness, sourness, bitterness, astringency and umami. In addition, aftertaste of bitterness, aftertaste of astringency and richness (persistence of umami) were also measured. Because the scores of sourness and astringency were lower than those of tasteless points, the tastes of sourness and astringency were not measured in PGT and SGT, while both PGT and SGT had similar attributes of aftertaste of astringency (*p* > 0.05). The bitterness and aftertaste of bitterness in PGT and SGT were near the tasteless point. PGT and SGT showed taste attributes of sweetness, saltiness, umami, richness and aftertaste of astringency, and no significant difference was observed in these taste attributes between PGT and SGT. Results of electronic tongue analysis indicated that both PGT and SGT had strong sweet and umami taste, but little astringent and bitter taste, and the process of pan-fire or steam had no significant effect on the taste of green teas (*p* > 0.05).

### 3.3. Effect of Fixation Methods on Chemical Compositions

**Figure 4:**
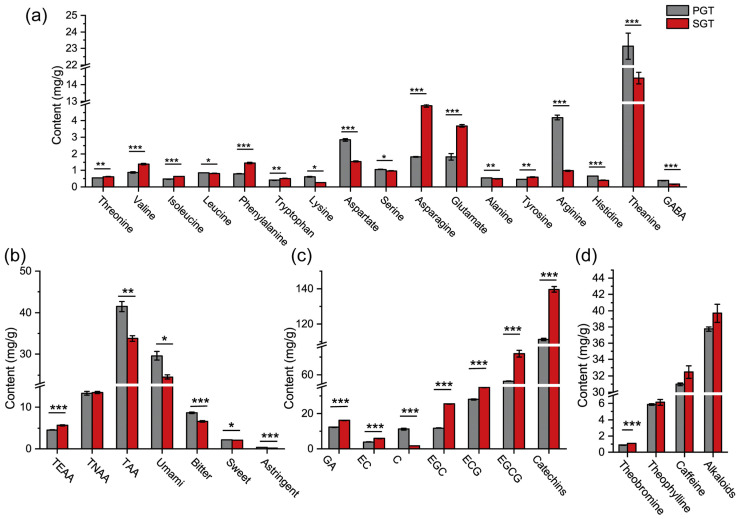
Effect of fixation methods on characteristic chemicals of green tea. (**a**,**b**) Contents of amino acids; (**c**) Contents of catechins; (**d**) Contents of alkaloids. PGT, pan-fired green tea; SGT, steamed green tea; TAA, total amino acids; TEAA, total essential amino acids; TNEAA, total non-essential amino acids; Thea, theanine; GABA, γ-aminobutyric acid; GA, gallic acid, EC, epicatechin; C, catechin; EGC, epigallocatechin; ECG, epicatechin gallate; EGCG, epigallocatechin gallate. * *p* < 0.05; ** *p* < 0.01; *** *p* < 0.001.

Characteristic chemical compositions including amino acids, catechins and alkaloids of PGT and SGT were shown in Figure 4. A total of 17 amino acids were detected in PGT and SGT, including 7 essential amino acids, 8 non-essential amino acids and 2 other non-protein amino acids (Figure 4a). Their content data were shown in Table S3. As shown in Figure 4b, the content of the total amino acids (TAA) was higher in PGT (41.47 mg/g) compared to SGT (33.79 mg/g) (*p* < 0.01), and SGT had higher total essential amino acids (TEAA) content (*p* < 0.001). Horanni and Engelhardt [25] have reported that the TAA content ranged from 8.37 to 46.8 mg/g in different green tea samples, which indicated that TAA contents of PGT and SGT both were at a relatively higher level. Pan-fire fixation with a high temperature and long time promotes the thermal hydrolysis of proteins, which may contribute to the higher level of amino acids in PGT. Additionally, amino acids are the precursors for the formation of many volatile compounds such as pyran and pyrrole by Maillard reactions [26]. Amino acids are known to have different tastes, including umami (aspartate, asparagine, glutamate, theanine), sweetness (threonine, serine, alanine), bitterness (valine, isoleucine, leucine, phenylalanine, tryptophan, lysine, tyrosine, arginine) and astringency (γ-aminobutyric acid) [27]. Amino acids in green teas are mainly responsible for umami taste. Theanine, asparagine, aspartate and glutamate contributing to the umami taste were the most abundant amino acids in PGT and SGT. In addition, as shown in Figure 4b, contents of umami, sweet, bitter and astringent amino acids at 29.61, 2.15, 8.67 and 0.38 mg/g in PGT, respectively, were all higher (*p* < 0.05) than those at 24.53, 2.08, 6.61 and 0.17 mg/g in SGT, respectively. However, compared to SGT, only slightly stronger umami and richness tastes were detected (Figure 3) in PGT (*p* < 0.05), which may be explained by the fact that the excess content of umami amino acids did not reach the threshold of taste change.

Phenols are the characteristic chemicals in green tea which have astringent taste and health benefits, and catechins account for approximately 70–80% of total tea polyphenol [28]. As shown in Figure 4c, the total content of five tested catechins (EC, C, EGC, ECG, EGCG) was significantly higher (*p* < 0.001) in SGT (139.68 mg/g) compared to that in PGT (111.36 mg/g). This result was in accordance with a previous study [29] which showed that the catechins content in PGT (85.07 mg/g) was lower compared to SGT (93.74 mg/g). EGCG was the most abundant catechin in PGT (56.58 mg/g) and SGT (72.03 mg/g), followed by ECG (27.97 mg/g in PGT, and 34.77 mg/g in SGT). Catechins EC, EGC, EC and EGCG all showed a higher content in SGT (*p* < 0.001). GA, which can be produced by hydrolysis of gallated catechins [30], also showed a higher content in SGT at 16.11 mg/g compared to PGT at 12.23 mg/g (*p* < 0.001). Steam treatment with a short time reduced the loss of catechins in green tea. Fan et al. [31] reported that long time thermal treatment promotes the degradation of catechins by oxidation, condensation, epimerization and hydrolysis reactions. Compared to the reported catechin content range of 70.16–141.54 mg/g [32], the levels of catechins which contribute to the astringency were not low in PGT and SGT. However, no astringency taste was measured by electronic tongue system (Figure 3), which may be explained by the low ratio of polyphenols to TAA (PP/AA). The ratio of PP/AA is regarded as an index reflecting the balance of phenols and free amino acids, and green teas with low PP/AA value (5.53–8.91) had high total quality scores [33]. With high contents of amino acids and low radio of PP/AA existing, PGT and SGT showed strong umami and little astringent taste.

Caffeine, theobromine and theophylline, which have bitter tastes, are the main alkaloids found in teas [28]. As shown in Figure 4d, there were no significant differences in the contents of total alkaloids, caffeine and theophylline (*p* > 0.05) between PGT and SGT (details in Table S3). Caffeine was the most abundant alkaloid in PGT (30.97 mg/g) and SGT (32.46 mg/g). Previous report [6] showed that caffeine contents in PGT and SGT were 2.50 and 2.27%, respectively. Significant difference in the content of theobromine was noted between PGT (0.88 mg/g) and SGT (1.07 mg/g). Caffeine with a stable cyclic structure cannot be degraded easily, which showed a relatively stable content after processing in six types of tea [34].

### 3.4. Multivariate Statistical Analysis of Biochemical Data

**Figure 5:**
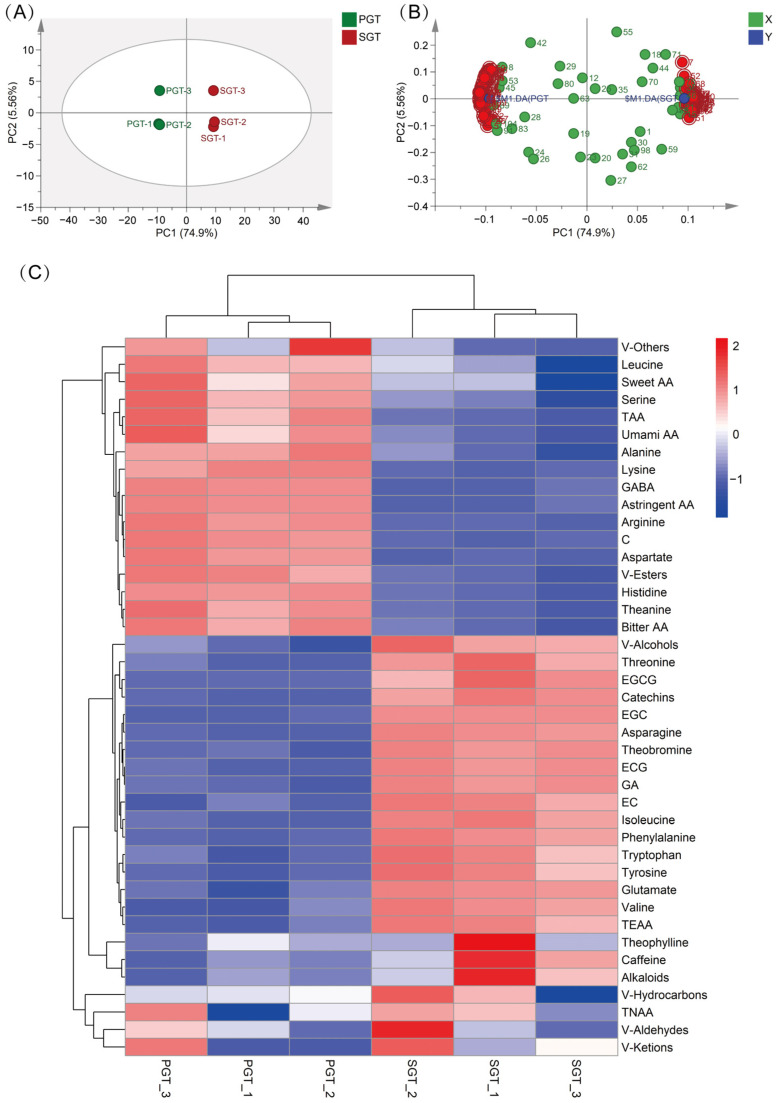
Comparison of PGT and SGT based on all biochemical compounds. (**A**) Score scatter plot of OPLS-DA analysis; (**B**) Loading scatter plot of OPLS-DA analysis; (**C**) Heatmap analysis. PGT, pan-fired green tea; SGT, steamed green tea; OPLS-DA, orthogonal partial least squares discriminant analysis; TAA, total amino acids; TEAA, total essential amino acids; TNEAA, total non-essential amino acids; Thea, theanine; GABA, γ-aminobutyric acid; GA, gallic acid; V-Alcohols, volatile alcohols; V-Aldehydes, volatile aldehydes; V-Ketones, volatile ketones; V-esters, volatile esters; V-Hydrocarbons, volatile hydrocarbons; V-Other, volatile others.

A total of 117 metabolites including volatile compounds, free amino acids, catechins and alkaloids were normalized by *Z*-score method to conduct OPLS-DA analysis. As shown in Figure 5A, PGT samples were clearly separated from SGT samples by PC1 that could explain 74.9% of the total variance. Based on the OPLS-DA loading plot (Figure 5B), VIP values and *p* values (Table S4), a total of 78 metabolites whose VIP > 1 and *p* < 0.05 were considered as the key metabolites contributing to the differentiation of PGT and SGT. These key metabolites included 55 volatiles, 16 amino acids, 6 phenols and 1 alkaloid. Heatmap (Figure 5C) was also prepared by metabolite data normalized by *Z*-score method, which could visually show the difference between PGT and SGT. The color scale from blue to red indicates the varying content of metabolites from relatively low to high. As shown in Figure 5C, PGT samples and SGT samples clustered, respectively, and these results were consistent with OPLS-DA analysis. All metabolites were clustered into two class, and 17 metabolites showed higher contents in PGT and 24 metabolites showed higher contents in SGT.

### 3.5. Effect of Fixation Methods on Lipid-Lowering Activity in a Zebrafish Larvae Model

**Figure 6:**
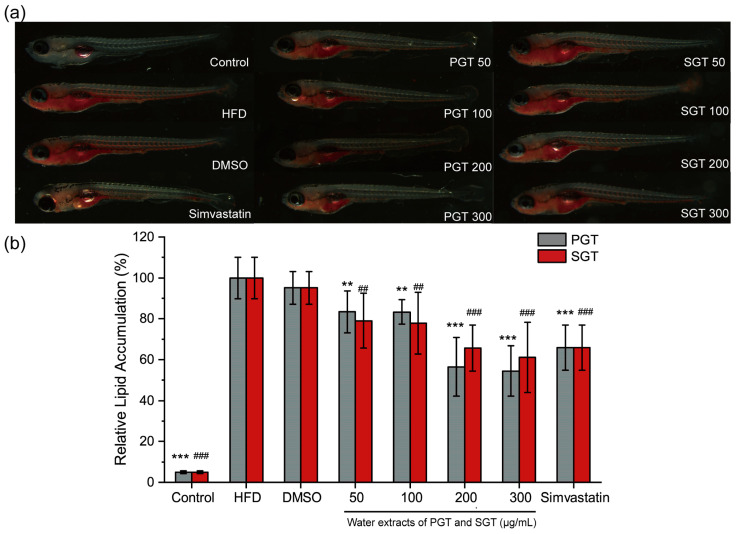
Assessment of lipid-lowering effects of PGT and SGT in a high-fat zebrafish larvae model. (**a**) Zebrafish larvae stained with oil red O and visualized under a microscope; (**b**) Relative lipid accumulation quantified with Image-pro plus software. PGT, pan-fired green tea; SGT, steamed green tea; HFD, high-fat diet. The two column charts of PGT and SGT in control, HFD, DMSO and simvastatin groups were produced based on the same data of each group. ‘*’ represents the significant difference between PGT group and HFD group; ** *p* < 0.01; *** *p* < 0.001; # represents the significant difference between SGT group and HFD group; ## *p* < 0.01; ### *p* < 0.001.

As shown in Figure 6a, after staining by oil red O, the lipid level of zebrafish larvae could be visually observed. Compared to the control group, the lipid levels of zebrafish larvae were significantly increased (*p* < 0.001) after a 48 h high-fat diet, which indicated the high-fat zebrafish larvae model was successfully established. Water extracts of PGT and SGT both showed the lipid-lowering effect. As shown in Figure 6a,b, water extract of PGT significantly reduced the lipid level by 16.61, 16.68, 43.51 and 45.53% at concentrations of 50, 100, 200 and 300 mg/L, respectively. Water extract of SGT also significantly reduced the lipid level by 20.94, 22.22, 34.38 and 38.89% at concentrations of 50, 100, 200 and 300 mg/L, respectively. The lipid-lowering effects of PGT and SGT were dose dependent. No significant difference was observed regarding the reduction in the lipid level between PGT and SGT at all concentrations (*p* > 0.05). The hypolipidemic drug simvastatin (0.04 mg/L) reduced the lipid level significantly by 34.17% (*p* < 0.001), and DMSO as a solvent control showed no significant effect on the lipid level at a concentration of 0.1% (*v*/*v*).

A previous study has reported that green tea extract significantly reduces visceral adiposity and plasma triglyceride content in zebrafish obesity models according to the activation of STAT pathway and the inhibition of CEPB pathway [35]. Green tea and catechins were reported to reduce the weight, hepatic lipids and white adipose tissue weights in high-fat diet animals [12]. Green teas show lipid-lowering effect through inhibiting the absorption and accumulation of lipids and promoting the energy expenditure, and EGCG as the most abundant catechins is considered to be the key contributor to the lipid-lowering effect of green tea [36]. EGCG can be combined with lipid and lipolytic enzymes to promote the lumina processes of emulsification, hydrolysis, micellar solubilization and subsequent uptake of lipids [36]. Moreover, experiments in vivo and vitro indicated that EGCG can inhibit the expression of genes and proteins which contributed to lipid accumulation, such as C/EBPα and PPAR-γ in adipogenesis, and fatty acid synthase and hydroxymethylglutaryl-CoA in lipogenesis [1]. Especially, previous studies reported that catechins and EGCG showed synergistic effects of caffeine on anti-obesity activities, and combination treatments displayed better results than individual treatments [13,37]. Therefore, it was suggested that high contents of caffeine and catechins may contribute to the lipid-lowering effect of PGT and SGT, and the precise influencing mechanism needs further investigation.

## 4. Conclusions

In this study, we investigated the effect of different fixation methods including pan-fire and steam on the aroma and taste qualities, characteristic chemical compounds and lipid-lowering activities of green teas. PGT and SGT could be distinguished by OPLS-DA based on both volatile compounds and all biochemical compounds. Volatile alcohols, hydrocarbons and esters were abundant in both PGT and SGT. SGT showed a higher content in volatile alcohols with green and floral aroma attributes, while PGT had higher content of volatile esters with fruity and sweet odor notes. Strong umami and sweetness, but little astringency and bitterness, were detected in PGT and SGT, and there was no significant difference between PGT and SGT in electronic tongue results. Overall, compared to each another, PGT had significantly higher contents of amino acids and SGT had significantly higher contents of catechins. Both PGT and SGT water extracts had effective lipid-lowering activities at all tested concentrations, and different fixation methods of pan-fire and steam showed no effect on the lipid-lowering activity of green tea. Generally, different fixation methods of pan-fire and steam showed significant effects on aroma and characteristic chemical compounds, but no effect on the taste and lipid-lowering activity.

## Data Availability

The data presented in this study are available in this article and supplementary materials.

## Supplementary Materials

The following supporting information can be downloaded at: https://www.mdpi.com/article/10.3390/foods11111582/s1, Table S1: Volatile compounds in PGT and SGT; Table S2: VIP values in OPLS-DA model and *p* values of volatiles compounds; Table S3: Contents of chemical compounds in PGT and SGT; Table S4: VIP values in OPLS-DA model and *p* values of chemical compounds.
