# Supplementary material for: Effect of Fixation Methods on Biochemical Characteristics of Green Teas and Their Lipid-Lowering Effects in a Zebrafish Larvae Model
Source: Foods. 2022 May 28;11(11):1582. doi: 10.3390/foods11111582 (PMC9180411; doi:10.3390/foods11111582)
Supplement: Supplementary file 1 [file foods-11-01582-s001.zip › foods-1743699-supplementary.pdf]

Supplementary Materials

Effect of Fixation Methods on Biochemical Characteristics of Green Teas and Their Lipid-Lowering Effects in a Zebrafish Larvae Model

This file contains the following tables:

Table S1. Volatile compounds in PGT and SGT.

Table S2. VIP values in OPLS-DA model and *p* values of volatiles compounds.

Table S3. Contents of chemical compounds in PGT and SGT.

Table S4. VIP values in OPLS-DA model and *p* values of chemical compounds.

Table S1. Volatile compounds in PGT and SGT.

ID	Name	Retention Index	CAS	Relative Content (%)	
				PGT	SGT
		Alcohols			
1	(*Z*)-3-Hexen-1-ol	868	928-96-1	1.86 ± 0.07	2.26 ± 0.48
2	1-Hexanol	860	111-27-3	-	3.14 ± 0.12
3	5-Methyl-2-heptanol	915	54630-50-1	-	1.27 ± 0.17
4	1-Octen-3-ol	969	3391-86-4	0.83 ± 0.09	1.5 ± 0.1
5	Benzyl alcohol	1036	100-51-6	1.26 ± 0.36	-
6	Myrcenol	1064	543-39-5	1.32 ± 0.11	-
7	1-Octanol	1059	111-87-5	0.76 ± 0.1	1.16 ± 0.11
8	(*E*)-Linalool oxide (furan)	1164	34995-77-2	1.52 ± 0.22	0.62 ± 0.44
9	Linalool	1082	78-70-6	14.21 ± 0.86	23.7 ± 0.98
10	Phenylethyl Alcohol	1136	60-12-8	1.7 ± 0.15	0.47 ± 0.33
11	(*S*)-3,7-Dimethyl-1-octanol	1130	68680-98-8	0.1 ± 0.01	-
12	(*Z*)-3-Nonen-1-ol	1167	10340-23-5	0.1 ± 0	0.09 ± 0.07
13	1-Nonanol	1159	143-08-8	-	0.55 ± 0.06
14	*trans*-2-Pinanol	1088	4948-29-2	-	1.93 ± 0.18
15	(*Z*)-Nerol	1228	106-25-2	0.17 ± 0.02	0.26 ± 0.03
16	Geraniol	1228	106-24-1	8.37 ± 1.02	4.93 ± 0.19
17	Cedrenol	1586	28231-03-0	-	2.85 ± 0.28
		Aldehydes			
18	Benzaldehyde	982	100-52-7	1.29 ± 0.28	1.58 ± 0.15
19	Nonanal	1104	124-19-6	1.25 ± 0.06	1.17 ± 0.39
20	Decanal	1204	112-31-2	0.55 ± 0.18	0.58 ± 0.08

21	β-Cyclocitral	1204	432-25-7	0.34 ± 0.02	0.48 ± 0.05
22	10-Undecenal	1293	112-45-8	0.18 ± 0.03	-
		Hydrocarbons			
23	1,3,5,7-Cyclooctatetraene	888	629-20-9	6.53 ± 1.87	6.24 ± 2.93
24	Myrcene	958	123-35-3	5.3 ± 0.73	3.13 ± 2.22
25	4-Methyl-3-(1-methylethylidene)-cyclohexene	1023	99805-90-0	0.42 ± 0.3	0.46 ± 0.12
26	o-Cymene	1042	527-84-4	0.44 ± 0.07	0.35 ± 0.1
27	D-Limonene	1018	5989-27-5	9.33 ± 3.16	10.91 ± 3.86
28	(*E*)-β-Ocimene	976	3779-61-1	0.6 ± 0.08	0.46 ± 0.1
29	(*Z*)-β-Ocimene	976	3338-55-4	0.9 ± 0.2	0.82 ± 0.13
30	y-Limonene	1013	499-97-8	0.71 ± 0.08	0.87 ± 0.24
31	Terpinolene	1052	586-62-9	0.39 ± 0.06	0.69 ± 0.6
32	1-Undecene	1105	821-95-4	0.3 ± 0.07	-
33	(*Z*)-4-Undecene	1123	821-98-7	0.45 ± 0.05	-
34	Azulene	1069	275-51-4	-	3.65 ± 0.43
35	Dodecane	1214	112-40-3	0.66 ± 0.19	0.74 ± 0.15
36	Benzocycloheptatriene	1251	264-09-5	0.29 ± 0.04	0.65 ± 0.08
37	1-Tetradecene	1403	1120-36-1	-	0.19 ± 0.02
38	Tetradecane	1413	629-59-4	0.71 ± 0.1	1.09 ± 0.11
39	Cedrene	1398	11028-42-5	0.28 ± 0.03	0.96 ± 0.09
40	Acenaphthylene	1460	208-96-8	0.08 ± 0.01	2.03 ± 0.24
41	Acenaphthene	1478	83-32-9	1.81 ± 0.12	-
42	Pentadecane	1512	629-62-9	0.89 ± 0.5	0.45 ± 0.08
43	β-Bisabolene	1500	495-61-4	1.04 ± 0.29	-
44	*cis*,*cis*-5,9-Tetradecadiene	1429	51255-62-0	0.46 ± 0.09	0.56 ± 0.03

45	Germacrene D	1515	23986-74-5	0.38 ± 0.05	0.25 ± 0.03
46	1-(o-Ethylphenyl)-1-phenyl-ethane	1715	18908-70-8	0.18 ± 0.02	0.31 ± 0.05
47	7-Methyl-heptadecane	1746	20959-33-5	0.81 ± 0.06	0.19 ± 0.03
48	Anthracene	1782	120-12-7	1.7 ± 0.11	0.4 ± 0.06
			Ketones		
49	2-Methyloctan-3-one	988	923-28-4	-	1.21 ± 0.36
50	Acetophenone	1029	98-86-2	0.13 ± 0.04	-
51	3,5-Octadien-2-one	968	38284-27-4	-	0.85 ± 0.18
52	Nona-3,5-dien-2-one	1068	80387-31-1	0.1 ± 0.03	0.55 ± 0.15
53	(*R,S*)-5-Ethyl-6-methyl-3E-hepten-2-one	1031	57283-79-1	0.39 ± 0.02	0.35 ± 0.01
54	Jasmone	1338	488-10-8	2.14 ± 0.18	0.91 ± 0.06
55	6,10-Dimethyl-5,9-undecadien-2-one	1420	689-67-8	0.89 ± 0.14	0.94 ± 0.02
56	(*E*)-β-Ionone	1457	79-77-6	0.34 ± 0.02	0.54 ± 0.05
57	2-Heptadecanone	1847	2922-51-2	0.14 ± 0.01	0.08 ± 0.02
58	Phytol	2045	150-86-7	0.85 ± 0.25	-
			Esters		
59	Pentyl acetate	884	628-63-7	-	0.33 ± 0.24
60	Ethyl hexanoate	984	123-66-0	3.01 ± 0.49	0.28 ± 0.1
61	cis-3-Hexenyl Acetate	992	3681-71-8	1.65 ± 0.03	-
62	Hexyl acetate	984	142-92-7	0.64 ± 0.4	0.99 ± 0.35
63	2-Ethylhexyl acetate	1118	103-09-3	0.22 ± 0.02	0.21 ± 0.03
64	(*E*)-3-hexen-1-yl butyrate	1191	53398-84-8	1.13 ± 0.13	0.72 ± 0.02
65	ethyl (*Z*)-4-octenoate	1191	34495-71-1	0.67 ± 0.1	-
66	Methyl salicylate	1281	119-36-8	-	0.55 ± 0.02
67	Butyl hexanoate	1183	626-82-4	1.83 ± 0.25	-
68	*trans*-2-Hexenyl butyrate	1191	53398-83-7	0.11 ± 0.02	0.45 ± 0.07

69	Ethyl caprylate	1183	106-32-1	4.39 ± 0.82	-
70	2-Ethylhexyl acrylate	1208	103-11-7	1.22 ± 0.25	1.46 ± 0.13
71	*cis*-3-Hexenyl-α-methylbutyrate	1226	53398-85-9	0.21 ± 0.03	0.26 ± 0
72	Hexyl 2-methylbutyrate	1218	10032-15-2	0.58 ± 0.09	1.2 ± 0.12
73	(2-Methyl-5-oxooxolan-2-yl) acetate	1226	57681-51-3	0.04 ± 0.01	0.27 ± 0.04
74	Ethyl nonylate	1282	123-29-5	0.16 ± 0	-
75	Methyl geranate	1252	2349-14-6	-	0.43 ± 0.03
76	*β*-Terpinyl acetate	1348	10198-23-9	-	0.28 ± 0.01
77	(*Z*)-3-hexen-1-yl caproate	1389	31501-11-8	1.69 ± 0.17	0.53 ± 0.02
78	Hexyl hexanoate	1381	6378-65-0	0.49 ± 0.06	0.65 ± 0.02
79	Ethyl caprate	1381	110-38-3	0.36 ± 0.09	-
80	2,2,4-Trimethyl-1,3-pentanediol diisobutyrate	1605	6846-50-0	0.64 ± 0.15	0.58 ± 0.06
81	Heptafluorobutyric acid hexadecyl ester	1733	6385-15-5	0.18 ± 0.06	-
82	Ethyl tetradecanoate	1779	124-06-1	0.22 ± 0.05	-
83	Isopropyl myristate	1814	110-27-0	0.55 ± 0.39	-
84	Monobutyl Phthalate	1828	131-70-4	0.36 ± 0.09	-
85	Butyl isobutyl phthalate	1973	17851-53-5	0.52 ± 0.16	0.06 ± 0.02
86	Ethyl palmitate	1978	628-97-7	1.78 ± 0.58	-
		Others			
87	Phthalan	1036	496-14-0	0.39 ± 0.1	1 ± 0.04
88	Tea pyrrole	1054	2167-14-8	0.37 ± 0.01	-
89	Dibenzofuran	1483	132-64-9	0.8 ± 0.14	0.39 ± 0.07
90	Nonanoic acid	1272	112-05-0	0.2 ± 0.04	-
91	9-Hexadecenoic acid	1976	2091-29-4	0.16 ± 0.07	-

PGT, pan-fired green tea; SGT, steamed green tea.

Table S2. VIP values in OPLS-DA model and *p* values of volatiles compounds.

ID	Name	VIP value	*P* value
**9**	**Linalool**	**5.903**	**0.001**
27	D-Limonene	2.870	0.679
**69**	**Ethyl caprylate**	**2.724**	**0.002**
**34**	**Azulene**	**2.274**	**0.000**
**16**	**Geraniol**	**2.137**	**0.009**
**2**	**1-Hexanol**	**1.942**	**0.000**
**17**	**Cedrenol**	**1.771**	**0.005**
**60**	**Ethyl hexanoate**	**1.698**	**0.013**
24	Myrcene	1.687	0.259
**40**	**Acenaphthylene**	**1.217**	**0.007**
**14**	**trans-2-Pinanol**	**1.203**	**0.004**
23	1,3,5,7-Cyclooctatetraene	1.199	0.916
**67**	**Butyl hexanoate**	**1.137**	**0.000**
**41**	**Acenaphthene**	**1.120**	**0.002**
**86**	**Ethyl palmitate**	**1.109**	**0.049**
**61**	**cis-3-Hexenyl Acetate**	**1.026**	**0.000**
6	Myrcenol	0.815	0.004
48	Anthracene	0.804	0.000
3	5-Methyl-2-heptanol	0.794	0.009
5	Benzyl alcohol	0.790	0.039
54	Jasmone	0.763	0.001
49	2-Methyloctan-3-one	0.759	0.041
10	Phenylethyl Alcohol	0.756	0.009
77	(Z)-3-hexen-1-yl caproate	0.720	0.010
43	β-Bisabolene	0.646	0.036
8	(E)-Linalool oxide (furan)	0.545	0.061
58	Phytol	0.530	0.040
51	3,5-Octadien-2-one	0.526	0.002
39	Cedrene	0.421	0.001
4	1-Octen-3-ol	0.419	0.002
65	ethyl (Z)-4-octenoate	0.414	0.001
72	Hexyl 2-methylbutyrate	0.388	0.004
47	7-Methyl-heptadecane	0.383	0.000
87	Phthalan	0.379	0.001
83	Isopropyl myristate	0.350	0.183
13	1-Nonanol	0.341	0.000
66	Methyl salicylate	0.338	0.001
42	Pentadecane	0.293	0.338
85	Butyl isobutyl phthalate	0.287	0.052
52	Nona-3,5-dien-2-one	0.284	0.044
33	(Z)-4-Undecene	0.282	0.007
31	Terpinolene	0.274	0.515
75	Methyl geranate	0.268	0.000

7	1-Octanol	0.260	0.018
62	Hexyl acetate	0.258	0.411
64	(E)-3-hexen-1-yl butyrate	0.255	0.010
89	Dibenzofuran	0.254	0.020
59	Pentyl acetate	0.241	0.188
1	(Z)-3-Hexen-1-ol	0.239	0.309
38	Tetradecane	0.234	0.021
88	Tea pyrrole	0.227	0.001
36	Benzocycloheptatriene	0.227	0.005
79	Ethyl caprate	0.224	0.028
84	Monobutyl Phthalate	0.220	0.030
68	trans-2-Hexenyl butyrate	0.215	0.018
18	Benzaldehyde	0.186	0.273
32	1-Undecene	0.184	0.029
76	β-Terpinyl acetate	0.170	0.000
70	2-Ethylhexyl acrylate	0.149	0.289
73	(2-Methyl-5-oxooxolan-2-yl) acetate	0.146	0.013
30	y-Limonene	0.146	0.410
19	Nonanal	0.143	0.818
82	Ethyl tetradecanoate	0.139	0.003
56	(E)-β-Ionone	0.125	0.008
90	Nonanoic acid	0.122	0.020
37	1-Tetradecene	0.115	0.000
81	Heptafluorobutyric acid hexadecyl ester	0.112	0.045
22	10-Undecenal	0.111	0.001
91	9-Hexadecenoic acid	0.103	0.037
78	Hexyl hexanoate	0.099	0.026
74	Ethyl nonylate	0.097	0.000
28	(E)-β-Ocimene	0.089	0.226
21	β-Cyclocitral	0.087	0.030
46	1-(o-Ethylphenyl)-1-phenyl-ethane	0.083	0.023
45	Germacrene D	0.079	0.045
50	Acetophenone	0.078	0.047
26	o-Cymene	0.076	0.317
25	4-Methyl-3-(1-methylethylidene)-cyclohexene	0.073	0.889
44	cis,cis-5,9-Tetradecadiene	0.068	0.201
11	(S)-3,7-Dimethyl-1-octanol	0.061	0.009
35	Dodecane	0.055	0.662
15	(Z)-Nerol	0.053	0.023
29	(Z)-β-Ocimene	0.053	0.631
80	2,2,4-Trimethyl-1,3-pentanediol diisobutyrate	0.048	0.599
55	6,10-Dimethyl-5,9-undecadien-2-one	0.044	0.602
57	2-Heptadecanone	0.038	0.018
71	cis-3-Hexenyl-α-methylbutyrate	0.033	0.093

53	(R,S)-5-Ethyl-6-methyl-3E-hepten-2-one	0.026	0.063
20	Decanal	0.023	0.871
63	2-Ethylhexyl acetate	0.006	0.820
12	(Z)-3-Nonen-1-ol	0.001	0.927

Table S3. Contents of chemical compounds in PGT and SGT.

Compounds	Contents (mg/g)	
	PGT	SGT
ECG	27.97 ± 0.27	34.77 ± 0.23
EGC	11.75 ± 0.16	25.41 ± 0.13
C	11.18 ± 0.48	1.62 ± 0.01
EGCG	56.58 ± 0.07	72.03 ± 1.97
EC	3.86 ± 0.14	5.85 ± 0.17
Catechins	111.36 ± 0.67	139.68 ± 1.68
GA	12.23 ± 0.16	16.11 ± 0.12
Theophylline	5.89 ± 0.1	6.15 ± 0.36
Caffeine	30.97 ± 0.16	32.46 ± 0.78
Theobromine	0.88 ± 0.01	1.07 ± 0.01
Alkaloids	37.74 ± 0.26	39.68 ± 1.09
Threonine	0.54 ± 0	0.62 ± 0.01
Valine	0.88 ± 0.05	1.38 ± 0.04
Isoleucine	0.47 ± 0	0.64 ± 0.01
Leucine	0.85 ± 0	0.82 ± 0.02
Phenylalanine	0.8 ± 0.01	1.44 ± 0.04
Tryptophan	0.41 ± 0.01	0.51 ± 0.01
Lysine	0.61 ± 0.02	0.26 ± 0
Aspartate	2.84 ± 0.09	1.54 ± 0.03
Serine	1.05 ± 0.01	0.97 ± 0.01
Asparagine	1.82 ± 0.02	4.9 ± 0.08
Glutamate	1.81 ± 0.2	3.69 ± 0.07
Alanine	0.55 ± 0	0.49 ± 0.01
Tyrosine	0.46 ± 0	0.59 ± 0.02
Arginine	4.19 ± 0.15	0.98 ± 0.03
Histidine	0.66 ± 0	0.4 ± 0.02
Theanine	23.14 ± 0.79	14.4 ± 0.35
GABA	0.38 ± 0	0.17 ± 0.01
TEAA	4.56 ± 0.07	5.67 ± 0.12
TNAA	13.38 ± 0.43	13.56 ± 0.23
TAA	41.47 ± 1.25	33.79 ± 0.68
Umami AA	29.61 ± 1.04	24.53 ± 0.49
Sweet AA	2.15 ± 0.02	2.08 ± 0.03
Bitter AA	8.67 ± 0.19	6.61 ± 0.16
Astrengent AA	0.38 ± 0	0.17 ± 0.01

AAs, amino acids; TEAA, total essential amino acids; TNEAA, total non-essential amino acids; TAA, total amino acids.

Table S4. VIP values in OPLS-DA model and *p* values of chemical compounds.

ID	Compounds	VIP value	*P* value
**74**	**Ethyl nonylate**	**1.135**	**0.000**
**61**	**cis-3-Hexenyl Acetate**	**1.135**	**0.000**
**93**	**EGC**	**1.135**	**0.000**
**76**	**β-Terpinyl acetate**	**1.135**	**0.000**
**110**	**Asparagine**	**1.134**	**0.000**
**117**	**GABA**	**1.134**	**0.000**
**88**	**Tea pyrrole**	**1.134**	**0.001**
**66**	**Methyl salicylate**	**1.133**	**0.001**
**2**	**1-Hexanol**	**1.133**	**0.000**
**94**	**C**	**1.133**	**0.001**
**92**	**ECG**	**1.133**	**0.000**
**97**	**GA**	**1.133**	**0.000**
**114**	**Arginine**	**1.132**	**0.000**
**105**	**Phenylalanine**	**1.132**	**0.000**
**107**	**Lysine**	**1.132**	**0.002**
**115**	**Histidine**	**1.131**	**0.000**
**100**	**Theobromine**	**1.130**	**0.000**
**108**	**Aspartate**	**1.130**	**0.000**
**103**	**Isoleucine**	**1.130**	**0.000**
**41**	**Acenaphthene**	**1.130**	**0.002**
**75**	**Methyl geranate**	**1.130**	**0.000**
**14**	**trans-2-Pinanol**	**1.128**	**0.004**
**17**	**Cedrenol**	**1.127**	**0.005**
**6**	**Myrcenol**	**1.127**	**0.004**
**96**	**EC**	**1.125**	**0.000**
**116**	**Theanine**	**1.124**	**0.000**
**48**	**Anthracene**	**1.124**	**0.000**
**13**	**1-Nonanol**	**1.123**	**0.000**
**111**	**Glutamate**	**1.122**	**0.000**
**33**	**(Z)-4-Undecene**	**1.122**	**0.007**
**102**	**Valine**	**1.122**	**0.000**
**47**	**7-Methyl-heptadecane**	**1.121**	**0.000**
**40**	**Acenaphthylene**	**1.121**	**0.007**
**34**	**Azulene**	**1.121**	**0.000**
**67**	**Butyl hexanoate**	**1.119**	**0.000**
**11**	**(S)-3,7-Dimethyl-1-octanol**	**1.118**	**0.009**
**22**	**10-Undecenal**	**1.117**	**0.001**
**113**	**Tyrosine**	**1.116**	**0.001**
**3**	**5-Methyl-2-heptanol**	**1.116**	**0.009**
**95**	**EGCG**	**1.116**	**0.000**
**37**	**1-Tetradecene**	**1.116**	**0.000**
**77**	**(Z)-3-hexen-1-yl caproate**	**1.116**	**0.010**
**39**	**Cedrene**	**1.115**	**0.001**

**65**	**ethyl (Z)-4-octenoate**	**1.115**	**0.001**
**9**	**Linalool**	**1.114**	**0.001**
**101**	**Threonine**	**1.114**	**0.001**
**54**	**Jasmone**	**1.112**	**0.001**
**106**	**Tryptophan**	**1.108**	**0.001**
**112**	**Alanine**	**1.108**	**0.001**
**69**	**Ethyl caprylate**	**1.105**	**0.002**
**60**	**Ethyl hexanoate**	**1.104**	**0.013**
**73**	**(2-Methyl-5-oxooxolan-2-yl) acetate**	**1.104**	**0.013**
**87**	**Phthalan**	**1.103**	**0.001**
**90**	**Nonanoic acid**	**1.101**	**0.020**
**51**	**3,5-Octadien-2-one**	**1.098**	**0.002**
**4**	**1-Octen-3-ol**	**1.097**	**0.002**
**82**	**Ethyl tetradecanoate**	**1.094**	**0.003**
**68**	**trans-2-Hexenyl butyrate**	**1.092**	**0.018**
**32**	**1-Undecene**	**1.087**	**0.029**
**84**	**Monobutyl Phthalate**	**1.086**	**0.030**
**79**	**Ethyl caprate**	**1.079**	**0.028**
**109**	**Serine**	**1.078**	**0.004**
**43**	**β-Bisabolene**	**1.077**	**0.036**
**72**	**Hexyl 2-methylbutyrate**	**1.073**	**0.004**
**36**	**Benzocycloheptatriene**	**1.073**	**0.005**
**5**	**Benzyl alcohol**	**1.072**	**0.039**
**58**	**Phytol**	**1.070**	**0.040**
**10**	**Phenylethyl Alcohol**	**1.066**	**0.009**
**50**	**Acetophenone**	**1.059**	**0.047**
**16**	**Geraniol**	**1.056**	**0.009**
**81**	**Heptafluorobutyric acid hexadecyl ester**	**1.054**	**0.045**
**64**	**(E)-3-hexen-1-yl butyrate**	**1.054**	**0.010**
**56**	**(E)-β-Ionone**	**1.052**	**0.008**
**49**	**2-Methyloctan-3-one**	**1.051**	**0.041**
**7**	**1-Octanol**	**1.048**	**0.018**
**86**	**Ethyl palmitate**	**1.043**	**0.049**
**52**	**Nona-3,5-dien-2-one**	**1.040**	**0.044**
85	Butyl isobutyl phthalate	1.040	0.052
**57**	**2-Heptadecanone**	**1.019**	**0.018**
38	Tetradecane	0.998	0.021
89	Dibenzofuran	0.998	0.020
15	(Z)-Nerol	0.998	0.023
46	1-(o-Ethylphenyl)-1-phenyl-ethane	0.995	0.023
78	Hexyl hexanoate	0.991	0.026
91	9-Hexadecenoic acid	0.980	0.037
104	Leucine	0.978	0.033
21	β-Cyclocitral	0.974	0.030
45	Germacrene D	0.936	0.045
8	(E)-Linalool oxide (furan)	0.924	0.061

99	Caffeine	0.909	0.057
53	(R,S)-5-Ethyl-6-methyl-3E-hepten-2-one	0.905	0.063
71	cis-3-Hexenyl-α-methylbutyrate	0.903	0.093
59	Pentyl acetate	0.883	0.188
83	Isopropyl myristate	0.830	0.183
24	Myrcene	0.741	0.259
44	cis,cis-5,9-Tetradecadiene	0.734	0.201
42	Pentadecane	0.729	0.338
26	o-Cymene	0.723	0.317
18	Benzaldehyde	0.696	0.273
62	Hexyl acetate	0.693	0.411
28	(E)-β-Ocimene	0.676	0.226
27	D-Limonene	0.665	0.679
98	Theophylline	0.630	0.377
1	(Z)-3-Hexen-1-ol	0.619	0.309
70	2-Ethylhexyl acrylate	0.604	0.289
55	6,10-Dimethyl-5,9-undecadien-2-one	0.588	0.602
30	y-Limonene	0.577	0.410
31	Terpinolene	0.564	0.515
20	Decanal	0.452	0.871
23	1,3,5,7-Cyclooctatetraene	0.441	0.916
29	(Z)-β-Ocimene	0.374	0.631
80	2,2,4-Trimethyl-1,3-pentanediol diisobutyrate	0.324	0.599
19	Nonanal	0.299	0.818
35	Dodecane	0.269	0.662
12	(Z)-3-Nonen-1-ol	0.167	0.927
63	2-Ethylhexyl acetate	0.138	0.820
25	4-Methyl-3-(1-methylethylidene)-cyclohexene	0.118	0.889
